# Mechanisms of Low Temperature Thickening of Different Materials for Deepwater Water-Based Drilling Fluids

**DOI:** 10.3390/gels10120789

**Published:** 2024-12-02

**Authors:** Zhongyi Wang, Jinsheng Sun, Kaihe Lv, Xianbin Huang, Zhenhang Yuan, Yang Zhang

**Affiliations:** 1 School of Petroleum Engineering, China University of Petroleum (East China), Qingdao 266580, China; 2 CNPC Engineering Technology R&D Co., Ltd., Beijing 102206, China

**Keywords:** deepwater drilling, water-based drilling fluid, low temperature gelation, flat rheology, drilling fluid construction method

## Abstract

During deepwater drilling, the low mudline temperatures and narrow safe density window pose serious challenges to the safe and efficient performance of deepwater water-based drilling fluids. Low temperatures can lead to physical and chemical changes in the components of water-based drilling fluids and the behavior of low temperature gelation. As a coarse dispersion system, water-based drilling fluid has a complex composition of dispersed phase and dispersing medium. Further clarification of low temperature gelation would be helpful in developing technical approaches to enhance the flat rheology performance of deepwater water-based drilling fluids. In this paper, different components are separated in order to comprehensively analyze the gelation behavior of different materials in water-based drilling fluids at low temperatures. In the first place, the rheological and hydrodynamic radius alterations of inorganic salts, bentonite, and additives in aqueous solutions were examined at low temperatures. The effects of inorganic salts, bentonite, and additives on the purified water system were investigated at low (4 °C)–normal (25 °C)–high (75 °C) temperatures. The low temperature gelation of different materials in pure water systems are fully clarified. The mud containing 4% bentonite with weak low temperature gelation commonly used in deepwater water-based drilling fluids was selected as the basic test system. Inorganic salts, additives, and solid-phase materials were added to the mud containing 4% bentonite. The effects of the interactions between different materials and bentonite particles on the low temperature gelation behavior of mud were analyzed. The higher the bentonite dosage, the stronger the low temperature gelation behavior of mud. The higher the addition of inorganic salts, the more serious the low temperature gelation behavior of mud. Inorganic salts should be avoided as much as possible to add too much. The low temperature gelation behavior of mud with low-viscosity additives is weak. However, the viscosity of mud with high-viscosity additives has a small change in viscosity with increasing temperature. The low temperature gelation of mud with the addition of solid-phase particulate materials with reactive groups on the surface is strong, and the low temperature gelation with the addition of inert particles is weak. This paper elucidates the low temperature gelation mechanism of bentonite, inorganic salts, additives, and solid-phase materials in deepwater water-based drilling fluids. The conclusion can also be used to guide the construction of a drilling fluid system, which is of great significance for the research and development of deepwater water-based drilling fluid additives and the safe and efficient performance of deepwater drilling fluids.

## 1. Introduction

In recent years, the world’s new oil and gas discoveries have mainly come from offshore, especially in deepwater and ultra-deepwater, and have shown a trend in which the deeper the water is, the greater the discovery [1,2,3]. The South China Sea is rich in geological resources of natural gas, and the total oil and gas resources in the South China Sea account for more than 1/3 of China’s total oil and gas resources; it is also one of the world’s four major seabed oil storage areas [4,5]. Deepwater and ultra-deepwater account for 76% of the sea area within the nine-dash line in the South China Sea, and deepwater oil and gas resources account for 70% of the total oil and gas resources in the South China Sea [6,7]. The South China Sea has huge potential for the development of deepwater oil and gas resources and is a key area for the subsequent development of China’s oil and gas resources [8].

With the continuous development of deepwater oil and gas resources in the South China Sea, the special environment of deepwater brings many challenges to safe and efficient drilling construction. The overlying rock layers in deepwater are equivalent to a portion being replaced by seawater compared to onshore; this results in lower pressures in the overlying rock layers than on land [9,10]. This feature results in lower rupture pressures in deepwater formations but not much variation in pore pressures, compared to on land; the difference between pore pressure and rupture pressure in deepwater formations is minimal. The narrow window of safe density makes it difficult to control the density of drilling fluids to ensure safe and smooth drilling operations. If the density of the drilling fluid is too low, it may cause formation fluids to intrude into the borehole, resulting in well wall collapse [11,12]. If the drilling fluid density is too high, the drilling fluid column pressure will exceed the formation rupture pressure, resulting in formation rupture and drilling fluid leakage [13]. The water temperature decreases as the depth of the seawater increases, and the temperature at the mudline of the deepwater seafloor is usually around 4 °C [14,15]. The low temperature of the seabed has a serious impact on the performance of the drilling fluids, mainly in the form of driving up the viscosity and yield value of the drilling fluid; even drilling fluid gelation can occur in severe cases [12,16]. This phenomenon can lead to greater circulating pressure depletion and higher column pressures, which can easily exceed the already narrow safe density window and exacerbate the deterioration of wellbore stability.

In the early days of deepwater drilling, high salt/polymer water-based drilling fluid systems were mostly used, which mainly include high salt/PHPA drilling fluid systems and high salt/polymer/polymer alcohol drilling fluid systems [17]. Low-viscosity polymers such as starch and PAC-LV (polyanionic cellulose) are mostly used to control system rheology. High concentrations of NaCl, together with the use of CaCl_2_, KCl, polymeric alcohols, and glycols, are used to provide adequate shale inhibition. In recent years, the strongly inhibited high-performance water-based drilling fluid system has been successfully applied in the Gulf of Mexico, South China Sea, Brazilian waters, and Colombian waters [18,19]. The key additives are low molecular amine-based polymers, with the use of xanthan gum, to improve low shear rate viscosity. The use of polyamines improves the thermal stability of xanthan gum. The system maintains good rheology in the 149 °C range [18,20]. However, as water depths continue to deepen, deepwater drilling faces more complex drilling environments, and the gelling of drilling fluids caused by the low temperatures in the mudline poses a significant challenge to the narrow safe density window in deepwater. In order to effectively control the flat rheological properties of water-based drilling fluids under deepwater low temperature conditions and avoid the drilling fluid gelation phenomenon at low temperatures, it is necessary to study the effect of low temperature on the performance of each component of water-based drilling fluids and to explain the mechanism of the effect of low temperature on the planar rheological properties of water-based drilling fluids [21]. This also lays a rigorous theoretical foundation for the development of deepwater water-based drilling fluid additives and the construction of drilling fluid systems.

Water-based drilling fluid is a dispersion system that uses water as the dispersion medium and drilling fluid materials as the dispersion phase [22]. The properties of the dispersed system are influenced by the dispersing medium and the dispersed phase [23]. The intricate composition of the components in water-based drilling fluid systems enables an in-depth analysis of their individual and collective mechanisms of action a challenging endeavor [24]. Therefore, this paper analyzes the changes in different drilling fluid materials in pure water systems and mud systems at low temperatures. By separating the different components, the changes in different materials at low temperatures are fully analyzed. Initially, the rheological and hydrodynamic radius changes in inorganic salts, bentonite, and additives were tested by subjecting them to low temperatures in water. The effect of different drilling fluid materials on the dispersion system was investigated under the conditions of low (4 °C)–normal (25 °C)–high (75 °C) temperature. On this basis, mud containing 4% bentonite was used as the basic test system. The effects of the interactions between inorganic salts, treating agents, solid-phase materials, and bentonite on the low temperature rheological behavior of deepwater water-based drilling fluids were explored. It serves as an inspiration for the theoretical study of deepwater water-based drilling fluids. To a certain extent, it can guide the material optimization of deepwater water-based drilling fluids.

## 2. Results and Discussion

### 2.1. Performance of Drilling Fluid Materials in Water at Low Temperature

**Figure 1:**
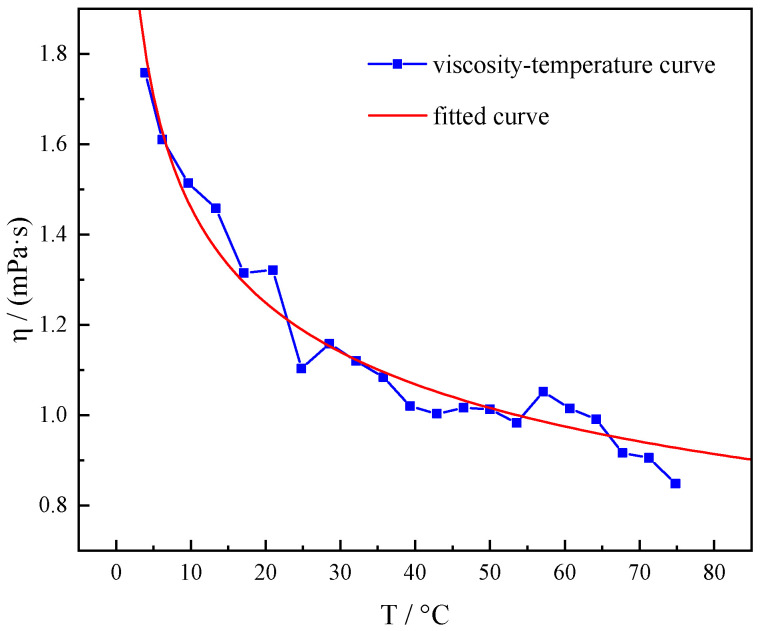
Viscosity–temperature curves of water at 4–75 °C.

Intermolecular thermal motion is diminished at low temperatures, resulting in water becoming less fluid and more viscous [25]. In order to study the effect of different drilling fluid materials on the low temperature gelation of water at low temperatures, it is necessary to clarify the change in the rheological properties of water itself with temperature. As clearly found in Figure 1, the viscosity of water was substantially increased from 0.849 mPa·s to 1.758 mPa·s during the process of temperature reduction from 75 °C to 4 °C. It indicates that the water molecules themselves are affected by the weakening of molecular thermal motion at low temperatures, which elevates the difficulty of flow, and the viscosity of the whole water system is substantially increased at low temperatures [26]. Water has a freezing point of 0 °C, and drilling fluid using water as a dispersing medium at mudline temperatures of 2–4 °C will exhibit low temperature gelation behavior. In order to analyze this phenomenon in depth, the viscosity–temperature curves of inorganic salts, bentonite, additives, and solid-phase materials in water were tested. The effect of various dispersing media in water on the low temperature rheological properties of the systems was analyzed.

#### 2.1.1. Inorganic Salt in Water

**Figure 2:**
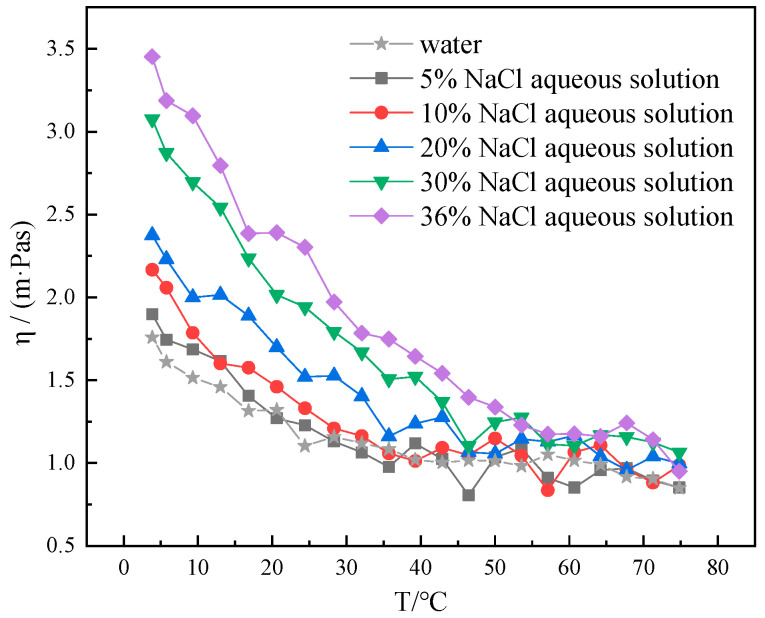
Viscosity–temperature curves of different concentrations (5%, 10%, 20%, 30%, and 36%) of NaCl aqueous solutions at 4–75 °C.

As can be seen from Figure 2, in the range of 4–75 °C, the viscosity–temperature curves of 5% NaCl aqueous solution and pure water showed a wide range of overlapping and interspersing, indicating that the addition of a small amount of NaCl had little effect on the viscosity of pure water. The viscosity of the aqueous NaCl solution increased with the increasing concentration of NaCl. Upon reaching a concentration of 20% NaCl in aqueous solution, a notable increase in viscosity can be observed at low temperatures. The viscosity of 20% NaCl aqueous solution at 4℃ increased from 1.758 mPa·s to 2.375 mPa·s compared to pure water. A significant increase in viscosity was also observed at 25 °C. The viscosity of 20% NaCl aqueous solution was elevated from 1.103 mPa·s to 1.519 mPa·s at 25 °C compared to pure water. With increasing temperature, the viscosity of 20%NaCl aqueous solution is very close to that of pure water at 75 °C. The concentration of the aqueous NaCl solution is increased to 30% or even 36% (saturated). At this point, an obvious viscosity thickening phenomenon can be observed under the low temperature and the normal temperature, and an obvious viscosity thickening phenomenon can be observed under the high temperature. At this concentration, the degree of viscosity increase at low temperatures is substantially higher than that at normal and high temperatures. This indicates that the low temperature viscosity-increasing behavior is stronger in aqueous solutions with higher NaCl concentrations. Water-based drilling fluid system with higher NaCl concentrations are more prone to gelation at low temperatures. The effect on the viscosity of pure water is minimal when the NaCl concentration is low, and the NaCl concentration should be controlled below 20% as much as possible.

**Figure 3:**
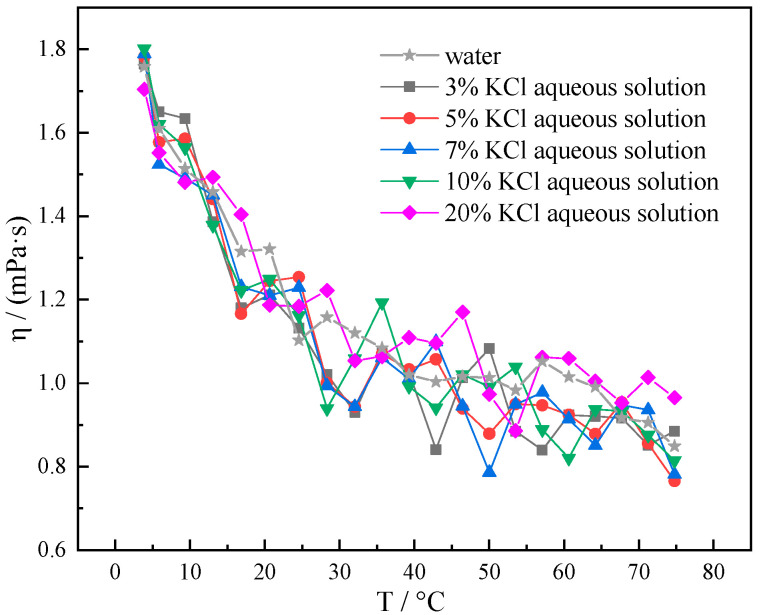
Viscosity–temperature curves of different concentrations of KCl (3%, 5%, 7%, 10%, and 20%) solutions at 4–75 °C.

As can be seen from Figure 3, within the temperature range of 4–75 °C, the amplitude of change in viscosity–temperature curves of aqueous KCl solutions with varying concentrations of KCl and pure water exhibits a relatively narrow range of variation. It indicates that the addition of a certain amount of KCl has little effect on the viscosity of pure water. Comparing with pure water, the viscosity of 10% KCl aqueous solution changed from 1.758 mPa·s to 1.801 mPa·s at 4 °C with a minimal change in good low temperature rheological properties. However, as the concentration of KCl increased, the viscous temperature curve of the aqueous KCl solution showed a large fluctuation above 25 °C. This situation is more dramatic in 10% KCl and 20% KCl aqueous solutions, increasing the difficulty of regulating the rheological properties of the system. The amount of KCl added to the pure water-based liquid system should be strictly controlled and not exceed 10%.

**Figure 4:**
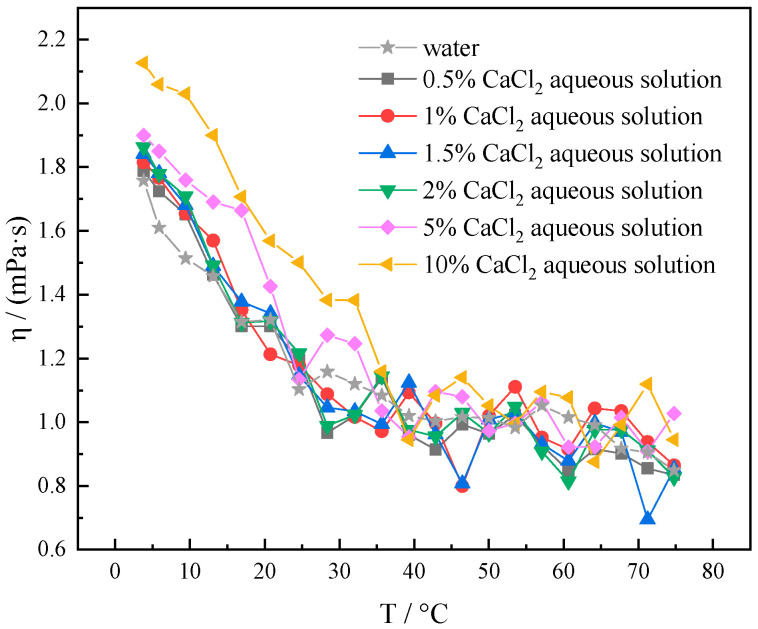
Viscosity–temperature curves of different concentrations of CaCl_2_ (0.5%, 1%, 1.5%, 2%, 5%, and 10%) solutions at 4–75 °C.

As can be seen from Figure 4, in the range of 4–75 °C, the concentration of 0.5–5.0% CaCl_2_ aqueous solution and pure water viscosity–temperature curves are both very close to each other. It indicates that the addition of a small amount of CaCl_2_ has little effect on the viscosity of pure water. The viscosity of 10% CaCl_2_ aqueous solution at 4 °C increased from 1.758 mPa·s to 2.127 mPa·s compared to pure water as the CaCl_2_ concentration increased. The viscosity was increased from 1.1581 mPa·s to 1.50 1mPa·s at 25 °C and from 0.8487 mPa·s to 0.9447 mPa·s at 75 °C. The viscosity enhancement rate reaches more than 20% at 4 °C and 50% at 25 °C, respectively. The effect of CaCl_2_ on the viscosity of pure water is small in the low concentration, the amount of CaCl_2_ should be kept below 10% at the time of use.

#### 2.1.2. Bentonite Particle in Water

Bentonite is a crucial component of drilling fluid systems, and its use in conjunction with other components can enhance drilling efficiency and mitigate costs [27]. However, in the face of the low temperature environment of deepwater drilling, bentonite particles of special lamellar structure and low temperature brought about by the molecular thermal movement changes, resulting in mud low temperature gelation properties [28,29]. In this subsection, the internal causes of the low temperature gelation phenomenon of muds are further analyzed by testing the viscosity, particle size and zeta potential of muds with different concentrations at 4–75 °C with respect to temperature.

**Figure 5:**
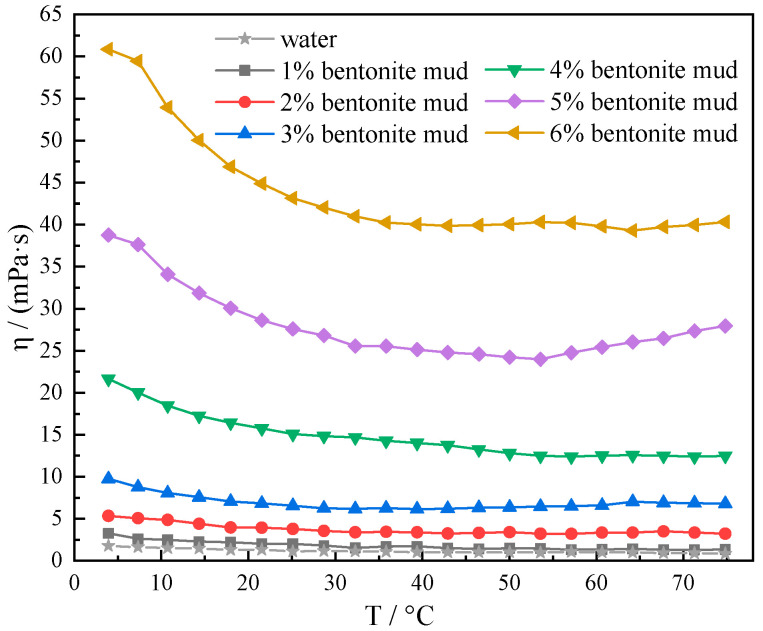
Viscosity–temperature curves of different concentrations (1%, 2%, 3%, 4%, 5%, and 6%) of mud at 4–75 °C.

From Figure 5, it is evident that within the temperature range of 4–75 °C, different concentrations of muds showed different degrees of viscosity thickening behavior at low temperatures. Moreover, the overall viscosity of the mud thickened as the addition of bentonite was elevated from 1% to 6, and the gelation behavior at low temperatures was enhanced. When the addition of bentonite was increased from 1% to 4%, the viscosity change rate is less than 50% in the temperature interval of 4–75 °C. Although the viscosity of the mud was thickened, the overall viscosity–temperature curve at this time was relatively smooth. The viscosity of mud is relatively stable with temperature when the bentonite dosage is from 1% to 4%. When the concentration of bentonite reaches 5% and 6%, the viscosity of mud increases significantly. The viscosity of the mud at 4 °C was 38.75 mPa·s for 5% bentonite dosage. The viscosity of the mud at 4 °C was 60.86 mPa·s for 6% bentonite dosage. At this time, the viscosity of mud at low temperature is too high to appear gelation phenomenon, which is very unfavorable to the drilling construction. In summary, the amount of bentonite added to deepwater water-based drilling fluids should be controlled between 2 and 4%.

**Figure 6:**
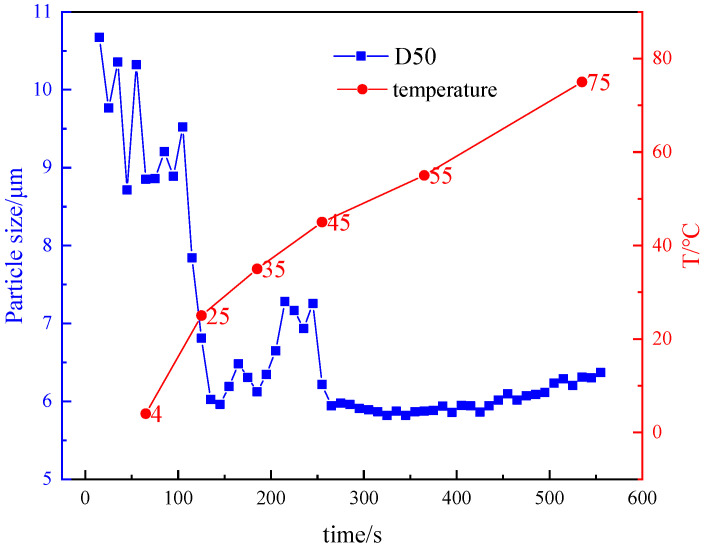
Changes in particle size of mud containing 4% bentonite at 4–75 °C.

As can be observed in Figure 6, the (Median particle size) D50 of mud containing 4% bentonite was 8.85 μm at 4 °C. The D50 of mud containing 4% bentonite was 6.8 μm at 25 °C. The D50 of mud containing 4% bentonite was 6.3 μm at 75 °C. The D50 of mud containing 4% bentonite decreases as the temperature decreases and the size of the bentonite particles increases at low temperatures. This is due to the fact that low temperatures inhibit the thermal movement of bentonite particles and reduce the hydration and dispersion capacity of bentonite particles. This leads to an increase in the interaction between bentonite particles, which form a strong lattice structure with “end-to-end” and “face-to-face” connections. This leads to an increase in particle size and low temperature gelation behavior.

**Figure 7:**
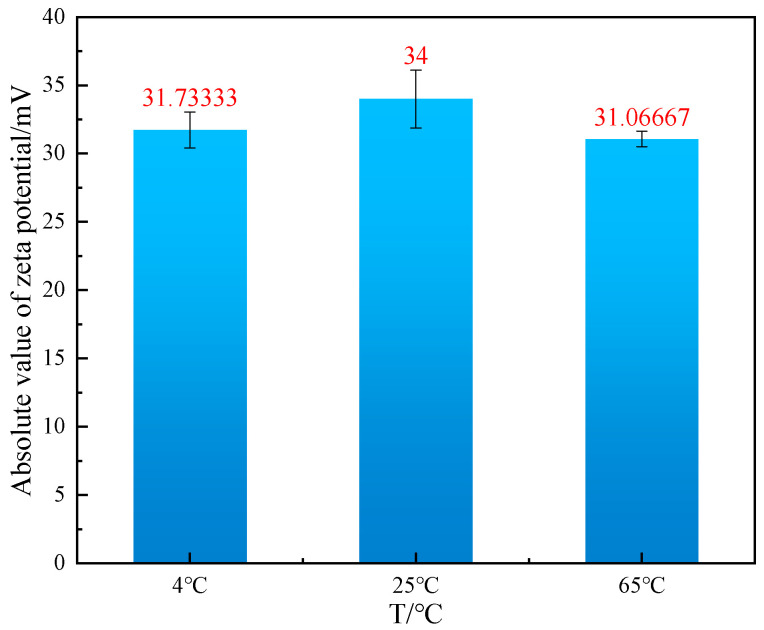
Changes in Zeta potential of mud containing 4% bentonite at 4–25–65 °C.

As can be observed in Figure 7, the absolute value of zeta potential of mud containing 4% bentonite is 31.73 mV at 4 °C, 34 mV at 25 °C, and 31.06 mV at 65 °C. The absolute value of zeta potential of mud containing 4% bentonite decreases at low temperatures, and the absolute value of zeta potential increases with increasing temperature. As the temperature increased, the absolute value of the zeta potential decreased once more. This phenomenon can be attributed to the fact that the low temperature environment impairs the diffusion bilayer ability of the bentonite particles’ surface. Consequently, the hydration film becomes thinner. The capacity of water molecules to penetrate the interior of bentonite is diminished, the dispersion of bentonite particles is reduced, and the absolute value of zeta potential is decreased. This, in turn, gives rise to augmented structural forces between the dispersed phases and complications in the flow of water molecules within the dispersed system, culminating in low temperature gelation behavior. In the construction of a deepwater water-based drilling fluid system, it is of paramount importance to exercise strict control over the quantity of bentonite present. The development of an additional additive is essential to mitigate the interaction of bentonite particles at low temperatures, thereby reducing the low temperature gelation behavior of mud. This will facilitate the enhancement of the amount of bentonite in the system, which will, in turn, augment the viscosity and yield value of the system.

#### 2.1.3. Additives in Water

**Figure 8:**
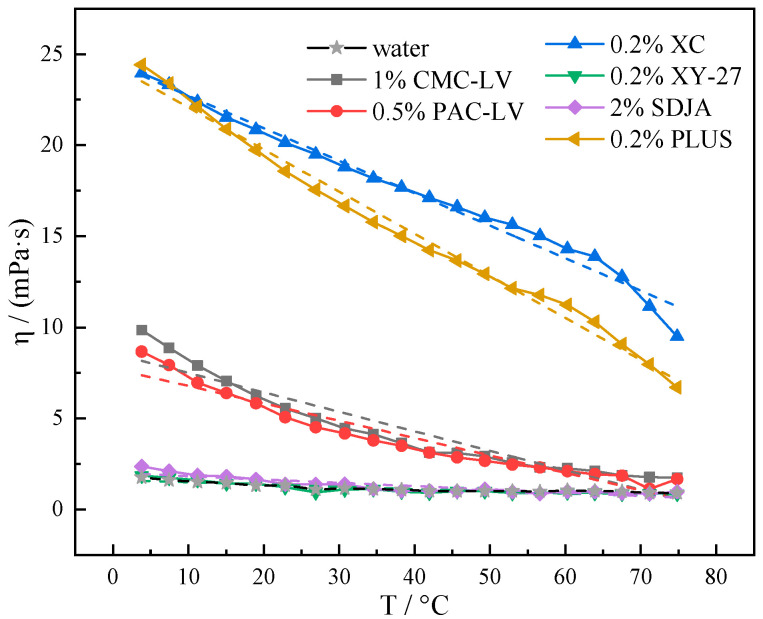
Viscosity changes in aqueous solutions of different additives at 4–75 °C.

It is difficult to analyze the effect of drilling fluid additives on the low temperature rheology of systems in complex systems. The temperature dependence of the viscosity of several additives of different properties in water itself is studied as a basis. The low-viscosity additives were selected as CMC-LV and PAC-LV, two filter loss reducers. For the high-viscosity additives, the thickening agent XC and the coating agent PLUS were selected. The inhibitor SDJA and viscosity reducer XY-27 were also selected. The different changes in different additives under the influence of low temperature were analyzed. On the basis of the viscosity–temperature curves of Figure 8, the distinct curves were subjected to linear regression analysis to derive the corresponding linear fit curves. The absolute values of the slopes of the fitted curves were then compared to analyze the rheological properties of the aqueous solutions of the different t additives and the data are shown in Table 1.

The absolute value of the slope of the linear fit to viscosity–temperature curve for pure water is only 0.00995. The viscosity–temperature curve patterns of several additives can be roughly categorized into three cases for comparison with pure water. The first type of low molecular weight polymers such as XY-27 and SDJA have linear fit slopes with absolute values of 0.0123 and 0.0181, respectively. The viscosity–temperature curves of the aqueous solutions of such additives do not change significantly in the range of 4–75 °C as compared to pure water curves. It is shown that in an aqueous environment, low molecular weight polymers have little effect on low temperature rheological properties of the system [30]. The second polymer additives as CMC-LV and PAC-LV had linear fit slopes with absolute values of 0.1069 and 0.0954, respectively. The viscosity–temperature curve of its aqueous solution varies greatly in the range of 4–75 °C compared with that of pure water, particularly exhibiting an increase in viscosity at low temperatures. These polymers CMC-LV and PAC-LV are able to increase the viscosity and shear strength of pure water systems at the appropriate concentration, but still have a distinctive “low temperature thickening, high temperature thinning” characteristic [29]. The third high-viscosity additives XC and PLUS have linear fit slopes with absolute values as high as 0.1784 and 0.2311, respectively. This high-viscosity additives increase viscosity greatly at low temperature, and the viscosity decreases more obviously with the increase in temperature. In the construction of deepwater water-based drilling fluid, low molecular weight polymer additives as XY-27 and SDJA should be used more often. Use some of the filter loss reducers and viscosity enhancers with insignificant viscosity increasing effect in small amounts. Strictly control the dosage or avoid the use of high-viscosity additives as much as possible.

### 2.2. Performance of Drilling Fluid Materials in Mud at Low Temperature

On the basis of pure water system, mud containing 4% bentonite was used as the basic test system to investigate the effects of interactions between inorganic salts, additives, solid-phase materials and bentonite on the low temperature rheological properties of mud.

#### 2.2.1. Inorganic Salt in Mud

NaCl, which has the most pronounced viscosity–temperature curve in aqueous solution, was chosen to be added to mud containing 4% bentonite The change in viscosity with temperature of mud containing 4% bentonite with NaCl additions of 10%, 20%, 30% and 36% were tested, and the viscosity–temperature curves were obtained and analyzed. 

**Figure 9:**
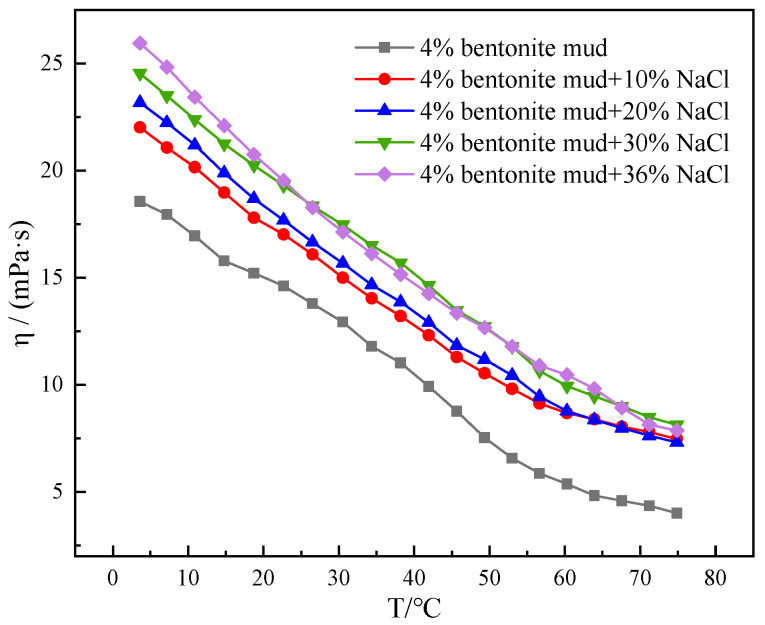
Viscosity–temperature curves for mud containing 4% bentonite with different NaCl concentrations (10%, 20%, 30%, and 36%) from 4 to 75 °C.

As can be observed in Figure 9, the viscosity of mud containing 4% bentonite with different concentrations of NaCl added were greatly enhanced. The viscosity of muds containing 4% bentonite increased with the rise of NaCl addition. It can be clearly observed that the low temperature gelation characteristics of the mud containing 4% bentonite become stronger and stronger at low temperatures with the increasing NaCl dosage, which is detrimental to the low temperature rheological control of the system. Although the viscosity of the mud containing 4% bentonite with NaCl increased at 75 °C, the viscosity of the mud containing 4% bentonite did not change much with the increasing NaCl dosage. This indicates that the increase in the amount of NaCl has less and less effect on the viscosity of the mud containing 4% bentonite system during the process of temperature increase. On the contrary, the rise in NaCl addition exacerbated the gelation of the water-based drilling fluid system at low temperatures, which was detrimental to the low temperature rheological regulation of the system. It shows that the amount of inorganic salts needs to be strictly controlled in mud-based drilling fluid systems.

#### 2.2.2. Additives in Mud

CMC-LV, PAV-LV, XC and PLUS, which have more pronounced viscosity–temperature curve changes in aqueous solution, were selected to be added to the mud containing 4% bentonite. The viscosity of mud containing 4% bentonite with 1% CMC-LV, 0.5% PAV-LV, 0.2% XC, or 0.2% PLUS were tested as a function of temperature, and viscosity–temperature curves were obtained and analyzed.

**Figure 10:**
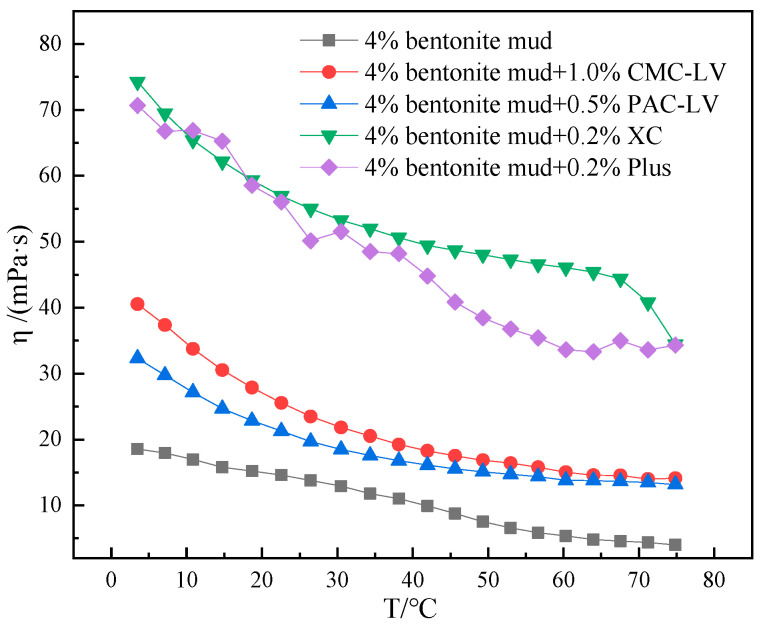
Viscosity–temperature curves of mud containing 4% bentonite with different treatments from 4 to 75 °C.

As can be observed from Figure 10, thickening of the viscosity of all mud occurred at 4–75 °C after the addition of different additives to the mud containing 4% bentonite. However, the viscosity–temperature curves of mud containing 4% bentonite with different additives also showed differences. The viscosity of mud containing 4% bentonite was 18.56 mPa·s at 4 °C and 4.01 mPa·s at 75 °C, with a viscosity improvement of 462.8% from 4 to 75 °C. Mud containing 4% bentonite has strong low temperature gelation and poor low temperature rheology. The viscosity of mud containing 4% bentonite with 1% CMC-LV added was 40.53 mPa·s at 4 °C and 14.13 mPa·s at 75 °C, giving a viscosity improvement of 286.8% from 4 to 75 °C. The viscosity of mud containing 4% bentonite with 0.5% PAC-LV added was 32.33 mPa·s at 4 °C and 13.16 mPa·s at 75 °C, giving a viscosity improvement of 245.6% from 4 to 75 °C. Compared to the mud containing 4% bentonite, the low temperature gelation effect of the mud containing 4% bentonite with 1% CMC-LV and 0.5% PAC-LV was significantly weaker. The mud containing 4% bentonite with additives maintains a high-viscosity at high temperatures. Compared to the mud with a viscosity of 4.01 mPa·s at 75 °C, the viscosities at 75 °C with the addition of the additives were 14.13 mPa·s and 13.16 mPa·s, respectively. The mud with additives has a moderate viscosity, which facilitates the introduction of other additives into the system when building the system. The viscosity of mud containing 4% bentonite with the addition of 0.2% XC was 74.3 mPa·s at 4 °C and 34.44 mPa·s at 75 °C, and the viscosity enhancement from 4 to 75 °C reached 215.7%. The viscosity of mud containing 4% bentonite with 0.2% Plus was 70.68 mPa·s at 4 °C and 34.32 mPa·s at 75 °C, and the viscosity enhancement from 4 to 75 °C reached 205.9%. It was clearly found that the viscosity enhancement of mud containing 4% bentonite with the addition of 0.2% XC and 0.2% Plus was significant, and the viscosity enhancement and gelation of mud was strong at low temperatures. However, the temperature-dependent rheological stability of these two high-viscosity additives in mud is stronger compared to the first two. The comparison shows that the rheological behavior of the different additives in mud is different from that occurring in the pure water system. This suggests that the interaction between bentonite particles and additives is enhanced at low temperatures in muds due to a more complex dispersion system. Relevant rheology modifiers should be developed that can effectively reduce the interaction between bentonite particles and additives at low temperatures to avoid the gelation behavior of low temperature drilling fluids.

**Figure 11:**
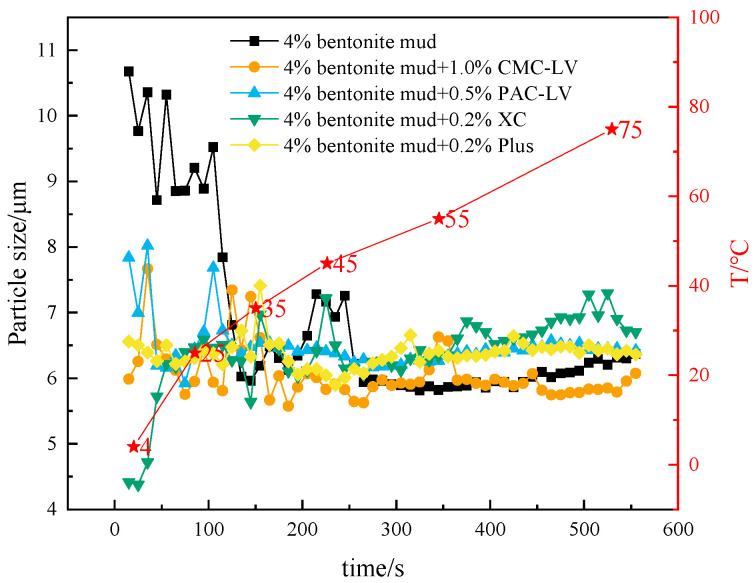
Changes in D50 from 4 to 75 °C for mud containing 4% bentonite with different treatments added.

The interaction of different additives with bentonite particles in mud was further analyzed under temperature variation from 4 to 75 °C. As can be observed in Figure 11, the D50 of all muds changed from 4 to 75 °C after the addition of different additives to the mud containing 4% bentonite It can be clearly found that the D50 of the mud containing 4% bentonite undergoes an elevation at low temperatures. This is due to the fact that low temperatures inhibit the thermal movement and hydration and dispersion of bentonite particles, leading to an increase in the interactions between the particles, which results in larger sizes of bentonite particles and even gelation. In contrast, the D50 change in mud with additives was significantly reduced at low temperatures. This suggests that polymer-based additives are beneficial to the rheological regulation of muds at low temperatures, regardless of the viscosity of the additives themselves. The inclusion of appropriate polymer additives is necessary when constructing deepwater water-based drilling fluid systems using mud as the base fluid. And as the temperature increases, the mud with polymer additives can still maintain good viscosity and yield value, which is also helpful to improve the rock-carrying capacity during the circulation of drilling fluid.

#### 2.2.3. Solid-Phase Material in Mud

The study of the role of different types of solid-phase materials in mud affected by low temperatures and thus on the rheology of the system was continued. The viscosity–temperature profiles of different muds at 4–75 °C were tested after adding 2% CaCO_3_, 2% FT, 2% SPNH, and 10% barite to a 4% mud.

**Figure 12:**
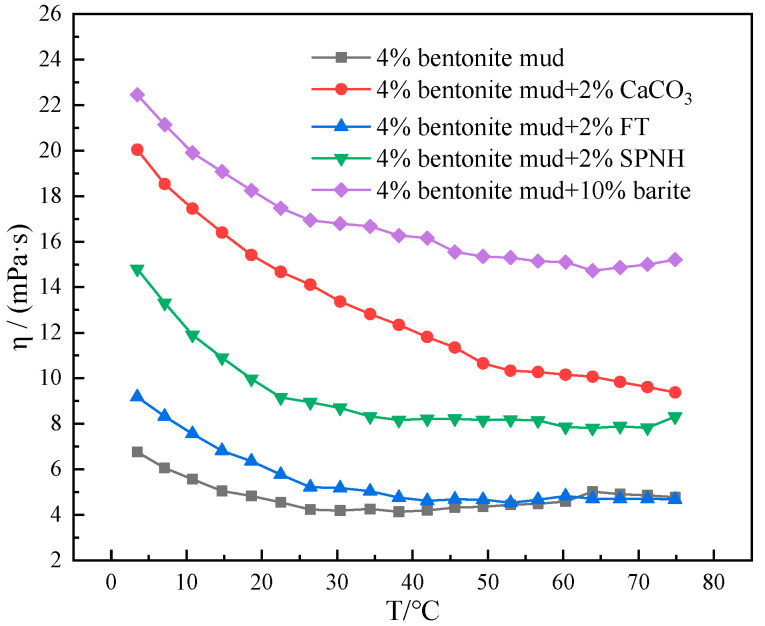
Viscosity–temperature curves of mud containing 4% bentonite with different materials at 4–75 °C.

As can be observed from Figure 12, there is a great difference in the viscosity–temperature curve performance of mud containing 4% bentonite at 4–75 °C with the addition of different solid-phase materials. The viscosity–temperature curves of mud containing 4% bentonite with the addition of 2% CaCO_3_, 2% SPNH and 10% barite showed similar trends from 4 to 75 °C. The viscosity thickening was significant at low temperatures and the viscosity decreased dramatically at increasing temperatures. Unlike these three, the viscosity profile of mud containing 4% bentonite with 2% FT is elevated at low temperatures due to the weakening of molecular thermal movement, while its viscosity profile gradually overlaps with that of mud as the temperature rises [31]. This is due to the weak water solubility of asphalt before the softening point, before reaching the corresponding softening point of weak water solubility on the viscosity of the system has little effect. Analyzing the viscosity–temperature curves of mud containing 4% bentonite with the addition of different solid-phase materials in the temperature interval of 4–75 °C indicates that micro- and nanomaterials and solid-phase particulate materials with reactive groups on their surfaces have stronger gelation effects at low temperatures, and relatively better low temperature rheological properties of inert particles. The type and amount of solid-phase materials should be strictly screened in the process of constructing deepwater water-based drilling fluid system.

### 2.3. Essentials for Building Water-Based Drilling Fluid Systems at Low Temperature

According to a variety of drilling fluid materials in water caused by viscosity, hydrodynamic radius and surface charge changes under the influence of low temperature. Summarized a variety of drilling fluid material itself in the water phase by the low temperature and the system caused by the law of change. Further taking the mud system as the background, the system change rules caused by inorganic salts, additives and solid-phase materials in mud subjected to low temperatures were investigated. The theoretical core of the construction of deepwater water-based drilling fluid system and the key points of different materials in the use of the system are clarified and summarized. The main points are as follows:

(1) The viscosity of water thickens considerably at low temperatures under the influence of weakened molecular thermal motion. In the environment of water-based drilling fluid, the existence of water as a dispersing medium, thickening or even gelation at low temperature is an unavoidable situation. When the drilling fluid system is constructed, the relevant additives should be strictly controlled and screened. And develop corresponding new additives to reduce the enhancement of low temperature gelation of water by other materials and additives through physicochemical means, and then improve the low temperature rheology of the system.

(2) The addition of large amounts of inorganic salts (saturated/multiple composite use) is unfavorable to the low temperature rheological control of water-based drilling fluids. The addition of inorganic salts should be strictly controlled when constructing deepwater water-based drilling fluid systems. Under the premise that solid-phase weighting materials have a more drastic effect on the low temperature rheology of the system, the density control of the system should be regulated by prioritizing the use of inorganic salts. At the same time, on the basis of meeting the hydrate inhibition performance, try to avoid adding too much inorganic salt.

(3) In the environment of deepwater deep wells, the high temperature at the bottom of the well brings about high temperature thinning of the drilling fluid, which weakens the rock-carrying capacity and suspension capacity. In order to maintain the yield value and viscosity of the drilling fluid, bentonite is an important and indispensable material in drilling operations. However, the low temperature gelation of muds in low temperature environments affects the low temperature rheology of deepwater systems. Excessive use of bentonite is detrimental to the construction of deepwater water-based drilling fluid systems. In order to cope with the low temperature gelation and the high temperature at the bottom of the well, the additions of bentonite should be controlled between 2 and 4%. In order to increase the amount of bentonite in the system, the development of rheology modifiers to reduce the gelation effect of bentonite particles at low temperatures is very necessary.

(4) The addition of polymer-based additives is beneficial to the low temperature rheological properties of mud. However, low molecular weight polymer additives should be used in the construction of deepwater water-based drilling fluid systems, and thickeners should be used in small quantities, and the amount of additives should be strictly controlled or the use of high-viscosity additives should be avoided as much as possible. Rheology modifiers with low molecular weights should be developed to attenuate the low temperature gelation caused by the interaction of other additives and bentonite. Low molecular weight or micro-nanoparticle types of anti-collapsing agents can also be used to replace the use of high molecular weight thickening agent in the system.

(5) The gelation effect of inert particles is weaker at low temperature, and the low temperature rheological performance is better. During the construction of deepwater water-based drilling fluid systems, the solid-phase materials used for blocking and lowering the filtration loss should be strictly selected to avoid influencing the low temperature rheological performance of the system. As for the weighting materials, due to the large amount of weighting materials used, the influence on the performance of the system is also greater. Should be in the priority of using inorganic salt aggravation premise, the subsequent use of solid-phase aggravating materials to make up to the corresponding stratum density. Reducing the use of solid-phase weighting materials also reduces the burden on the suspension capacity of the system.

### 2.4. Construction and Performance Evaluation of Deepwater Water-Based Drilling Fluid System

On the basis of the mechanism study, relevant additive materials were optimized to construct a deepwater water-based drilling fluid system with temperature resistance up to 180 °C and good low temperature rheological properties. The system is formulated as follows: 4% bentonite + 1% low-viscosity filter loss reducer + 0.25% rheology modifier + 2% hydrophobic inhibitor + 2% CaCO_3_ + 2% FT + 2% lubricant + 20% NaCl + barite weighted to 1.5 kg/m^3^.

From the experimental results in Table 2 and Table 3, it can be clearly found that the drilling fluid system before and after aging has stable rheological properties, low filtration and good base performance. Parameter ratios of drilling fluid system before aging: AV_4°C_:AV_25°C_ < 1.30, PV_4°C_:PV_25°C_ < 1.20, YP_4°C_:YP_25°C_ < 1.20. Parameter ratios of the drilling fluid system after aging: AV_4°C_:AV_25°C_ < 1.10, PV_4°C_:PV_25°C_ < 1.10, YP_4°C_:YP_25°C_ < 1.20. It is shown that the preferred drilling fluid system has good flat rheological properties [16].

## 3. Conclusions

The effects of low temperature on the components of water-based drilling fluids were investigated by the controlled variable method, combined with viscosity–temperature curves, zeta potential, and dynamic particle size experimental analysis. The fundamental principles underlying the formulation of water-based drilling fluids for deepwater wells and the characteristics of different drilling fluid materials were elucidated. The concept of constructing deepwater water-based drilling fluid systems for deepwater wells was established, with mud as the base fluid, low-viscosity polymer additives to regulate the system performance being preferred, the cooperation of inert solid-phase materials for regulating the loss of filtration, inorganic salts in conjunction with solid-phase aggravating materials, and the introduction of new types of rheology modifiers and anti-collapse materials.

## 4. Materials and Methods

### 4.1. Material and Sample Preparation

Materials include Bentonite (industrial grade), Sodium Chloride (NaCl, 99.5 wt%), Potassium Chloride (KCl, 99.5 wt%), Calcium Chloride (CaCl_2_, 99.5 wt%), Carboxymethyl Cellulose (CMC-LV, industrial grade), Polyanionic Cellulose (PAC-LV, industrial grade), High-viscosity Inhibitor (PLUS, industrial grade), Xanthan Gum (XC, industrial grade), Polyamine (SDJA, industrial grade), viscosity reducing agent (XY-27, industrial grade), ultrafine calcium carbonate (CaCO_3_, industrial grade), bitumen (FT, industrial grade), lignite resin (SPNH, industrial grade), barite (industrial grade), anhydrous sodium carbonate (Na_2_CO_3_, 99.5 wt%) low-viscosity filter loss reducer (Laboratory self-made), rheology modifier (Laboratory self-made), hydrophobic inhibitor (Laboratory self-made), and lubricant (Laboratory self-made).

Mud is configured as follows: add 400 mL of distilled water in a high stirring cup, then add a certain amount of bentonite and a certain amount of Na_2_CO_3_ (3 wt% of the amount of bentonite added) in the distilled water, use a high-speed inverter mixer to mix at high speed (6000 r/min) for 20 min, stop twice to scrape off the clay adhering to the wall of the cup, and place it in a closed container for 24 h. According to this method, the mud with bentonite additions of 1%, 2%, 3%, 4%, 5%, and 6% were configured.

Different types and concentrations of aqueous saline solutions and aqueous solutions of additives are configured. Among them, the NaCl solutions were 5%, 10%, 20%, 30% and 36%. The KCl solutions were 3%, 5%, 7%, 10%, and 20%. The CaCl_2_ solutions were 0.5%, 1.0%, 1.5%, 2.0%, 5%, and 10%. Additive solutions were 1.0% CMC-LV, 0.5% PAC-LV, 0.2% PLUS, 0.2% XC, 0.2% XY-27, and 2% SDJA.

Test muds were configured by adding different types and concentrations of salts, additives and solid-phase materials to the mud containing 4% bentonite. Configuration of mud with NaCl additions of 10%, 20%, 30% and 36%. Configuration of different mud containing 4% bentonite containing 1.0% CMC-LV, 0.5% PAC-LV, 0.2% PLUS, 0.2% XC, 2% FT, 2% CaCO_3_, 2% SPNH and 10% barite, respectively.

### 4.2. Rheological Variation at Low Temperature

The samples tested are as follows. Aqueous solutions of NaCl at concentrations of 5%, 10%, 20%, 30% and 36%. Aqueous solutions of KCl at concentrations of 3%, 5%, 7%, 10%, and 20%. CaCl_2_ aqueous solution at concentrations of 0.5%, 1.0%, 1.5%, 2.0%, 5%, and 10%. Mud with a bentonite content of 1%, 2%, 3%, 4%, 5%, and 6%. Additive solution with 1.0% CMC-LV, 0.5% PAC-LV, 0.2% PLUS, 0.2% XC, 0.2% XY-27, and 2% SDJA. Muds containing 4% bentonite with NaCl additions of 10%, 20%, 30% and 36%. Mud containing 4% bentonite with drilling fluid material additions of 1.0% CMC-LV, 0.5% PAC-LV, 0.2% XY-27, 0.2% PLUS, 0.2% XC, 2% FT, 2% CaCO_3_, 2% SPNH, and 10% barite. The temperature interval tested was 4–75 °C. Moreover, 4 °C is the mudline temperature for most deepwater blocks, and 75 °C is the temperature at which the mud circulates out of most wellheads in the field. A HAAKE rotational rheometer (HAKKE MARS60, Berlin, Germany) was used with an optional sleeve module using rotational shear temperature scanning. The angular velocity of the test was 170 s^−1^ and the test time was 30 min. The viscosity–temperature curves of the test liquids in the range of 4–75 °C were obtained.

### 4.3. Particle Size Variation at Low Temperature

The test samples are as follows: mud containing 4% bentonite and mud containing 4% bentonite with drilling fluid material additions of 1.0% CMC-LV, 0.5% PAC-LV, 0.2% XY-27, 0.2% PLUS, and 0.2% XC. The temperature interval tested was 4–75 °C. Using the Focused Beam Reflectometer (Mettler FBRM, Zurich, Switzerland), particle size variations can be tracked and tested in real time throughout the process. The test time was programmed to be 30 min with a test interval of 10 s. Temperature control was by means of a variable temperature thermostat. The particle size versus temperature curve of the test liquid at 4–75 °C was obtained.

### 4.4. Zeta Potential Variation at Low Temperature

The test sample was mud containing 4% bentonite. The test temperatures were 4 °C, 25 °C and 65 °C. The zeta potential of different liquids at different temperature points was tested using a Zeta Potential Analyser (Nano ZS, Malvern, UK) using the electrophoretic light scattering method technique. The strength of hydration on the surface of the dispersed phase in the tested liquids was analyzed by the change in zeta potential.

### 4.5. Drilling Fluid System Performance Testing

The deepwater water-based drilling fluid system (WBDF) was constructed with the developed low-viscosity filter loss reducer, rheology modifier, hydrophobic inhibitor, and lubricants under the premise of preferring other drilling fluid additives. The preferred drilling fluid system is as follows in Table 4:

The rheological performance was tested by using a viscometer (ZNN-D6B, Tongchun Petroleum Instrument, Qingdao, China). The drilling fluid system was aged at 180 °C for 16 h by using a high temperature roller heating oven. Before and after aging, a constant temperature and humidity chamber were used to control the temperature and a ZNN-D6B viscometer was used to test the parameters of the drilling fluid system (θ600, θ300) at 4 °C, 25 °C, and 50 °C. The apparent viscosity (AV), plastic viscosity (PV), and yield value (YP) of BF at 4 °C, 25 °C, and 50 °C were also calculated according to the API standard. The parameter ratios of AV, PV, and YP at 4 °C to 25 °C and 4 °C to 50 °C are also compared.

AV = 0.5·θ600(1)

PV = θ600 − θ300(2)

YP = 0.5·(θ300 − PV)(3)

where AV is apparent viscosity (mPa·s), PV is plastic viscosity (mPa·s), and YP is yield value (Pa).

The amount of medium pressure filtration (FL_1_) of the drilling fluid system before and after aging at 180 °C/16 h was determined using a medium pressure filtration meter (ZY-LS3, Tongchun Petroleum Instrument, Qingdao, China) with a test time of 7.5 min and a pressure difference of 100 psi. The high temperature and high pressure filtration (FL_2_) of drilling fluid after aging at 180 °C/16 h was tested by a high temperature and high pressure filtration tester (HH-260, Tongchun Petroleum Instrument, Qingdao, China) with a test time of 30 min and a pressure difference of 600 psi. Both of two were also calculated according to the API standard.

FL_API_ =FL_1_·2 (4)

FL_HTHP_ = FL_2_·2 (5)

where FL_API_ is medium pressure filtration (mL), FL_HTHP_ is high temperature and high pressure filtration (mL).

## Figures and Tables

**Table 1:** Linear fit curve data.

Additives	Linear Fit Curve	Absolute Value of the Slope of the Linear Fit
water	y = −0.00995x + 1.56272	0.00995
1% CMC-LV	y = −0.1069x + 9.5664	0.1069
0.5% PAC-LV	y = −0.0954x + 7.7330	0.0954
0.2% XC	y = −0.1784x + 24.4961	0.1784
0.2% PLUS	y = −0.2311x + 24.3801	0.2311
2% SDJA	y = −0.0181x + 2.0079	0.0181
0.2% XY-27	y = −0.0123x + 1.6260	0.0123

**Table 2:** General properties of drilling fluid systems.

Conditions	Density/ g/cm^3^	T/°C	AV/ mPa·s	PV/ mPa·s	YP/ Pa	θ6/θ3	FL_API_/ mL	FL_HTHP_/ mL
Before ageing	1.5	4	33	24	9	7/6	4	-
		25	27	20	8	6/5		
		50	24	15	9	8/7		
After 180 °C/16 h ageing	1.5	4	61	48	13	14/10	8	18.8
		25	58	46	12	11/10		
		50	36	28	8	10/9		

**Table 3:** Parameter ratios for different drilling fluid systems.

Parameter Ratios	Before Aging	After Aging
AV_4°C_:AV_25°C_	1.22	1.05
AV_4°C_:AV_50°C_	1.38	1.69
PV_4°C_:PV_25°C_	1.2	1.04
PV_4°C_:PV_50°C_	1.6	1.71
YP_4°C_:YP_25°C_	1.125	1.08
YP_4°C_:YP_50°C_	1	1.625

**Table 4:** Composition of the WBDFs.

Component	Function	Mass Fraction/wt%
bentonite	filtrate reducer and viscosifier	4
low-viscosity filter loss reducer	filtrate reducer and viscosifier	1
rheology modifier	viscosifier	0.25
hydrophobic inhibitor	shale inhibitor	2
CaCO_3_	plugging agent	2
FT	plugging agent	2
lubricants	Lubricants	2
NaCl	hydrate inhibitor	20

Note: drilling fluid systems were weighted to 1.5 g/cm^3^ by barite.

## Data Availability

Data underlying the results presented in this paper are not publicly available at this time but may be obtained from the corresponding authors upon reasonable request.
